# In diverse conditions, intrinsic chromatin condensates have liquid-like material properties

**DOI:** 10.1073/pnas.2218085120

**Published:** 2023-04-24

**Authors:** Bryan A. Gibson, Claudia Blaukopf, Tracy Lou, Lifeng Chen, Lynda K. Doolittle, Ilya Finkelstein, Geeta J. Narlikar, Daniel W. Gerlich, Michael K. Rosen

**Affiliations:** ^a^Department of Biophysics, University of Texas Southwestern Medical Center, Dallas, TX 75390; ^b^HHMI, University of Texas Southwestern Medical Center, Dallas, TX 75390; ^c^Institute of Molecular Biotechnology of the Austrian Academy of Sciences, Vienna BioCenter, 1030 Vienna, Austria; ^d^Department of Biochemistry and Biophysics, University of California, San Francisco, CA 94158; ^e^Department of Molecular Biosciences, University of Texas at Austin, Austin, TX 78712; ^f^Institute for Cellular and Molecular Biology, University of Texas at Austin, Austin, TX 78712; ^g^Center for Systems and Synthetic Biology, University of Texas at Austin, Austin, TX 78712

**Keywords:** chromatin, phase separation, biomolecular condensate

## Abstract

Nuclear DNA in eukaryotes is wrapped around histone proteins to form nucleosomes on a chromatin fiber. Dynamic folding of the chromatin fiber into loops and variations in the degree of chromatin compaction regulate essential processes such as transcription, recombination, and mitotic chromosome segregation. Our understanding of the physical properties that allow chromatin to be dynamically remodeled even in highly compacted states is limited. Previously, we reported that chromatin has an intrinsic capacity to phase separate and form dynamic liquid-like condensates, which can be regulated by cellular factors [B. A. Gibson *et al.*, *Cell* **179**, 470–484.e421 (2019)]. Recent contradictory reports claim that a specific set of solution conditions is required for fluidity in condensates that would otherwise be solid [J. C. Hansen, K. Maeshima, M. J. Hendzel, *Epigenetics Chromatin* **14**, 50 (2021); H. Strickfaden *et al.*, *Cell* **183**, 1772–1784.e1713 (2020)]. We sought to resolve these discrepancies, as our ability to translate with confidence these biophysical observations to cells requires their precise characterization. Moreover, whether chromatin assemblies are dynamic or static affects how processes such as transcription, loop extrusion, and remodeling will engage them inside cells. Here, we show in diverse conditions and without specific buffering components that chromatin fragments form phase separated fluids in vitro. We also explore how sample preparation and imaging affect the experimental observation of chromatin condensate dynamics. Last, we describe how liquid-like in vitro behaviors can translate to the locally dynamic but globally constrained chromatin movement observed in cells.

## Significance

The organization of eukaryotic chromatin is important in many nuclear processes. Recent studies have shown that chromatin fragments can self-assemble by phase separation into micron-scale structures in the presence of salt in vitro. There are discrepancies regarding whether these structures generally have liquid-like or solid-like behaviors, an important distinction in considering how processes such as transcription and chromosome remodeling by loop extrusion can occur in cells. Here, we resolve conflicting reports by demonstrating that chromatin condensates have liquid-like behaviors in diverse solution conditions and describing aspects of sample handling that can lead to artifactual solid-like behaviors. Our data suggest how chromatin can be dynamic on short length scales but restrained on long length scales, as observed in cells.

To maintain integrity during mitosis and fit into the nucleus, the eukaryotic genome must undergo substantial compaction (1). Chromatin is compacted by affinity-based interactions within the fiber and motor-driven extrusion of dynamic loops by protein complexes of the structural maintenance of chromosome (SMC) family. Together, these activities regulate many essential functions, including transcription, recombination, DNA repair, and chromosome segregation (2–5).

Individual genomic loci are constrained to move only within a locally defined region inside the nucleus, controlled by interchromatin interactions, physical crosslinks induced by macromolecular complexes, and attachment of chromatin to static nuclear structures (6–8). A detailed account of the physical mechanisms that package the genome is critical, given the importance of spatial organization in regulating DNA-templated processes such as transcription, DNA replication, and DNA repair (9, 10).

In a previous report, we described how chromatin has an intrinsic capacity to phase separate, producing liquid-like condensates with cell-like DNA density (11). Among other advances, this work shed light on the physical mechanism underlying a well-described assay for chromatin self-assembly, historically performed by adding superphysiological concentrations of divalent cation alone (12, 13). These intrinsic chromatin condensates, which refers here to factor-independent nucleosome-driven phase separation, can be regulated by cellular factors in kind with their functions in genome regulation (11, 14). We suggested that interchromatin interaction through intrinsic condensation could represent a “ground state” for chromatin organization, molded or disrupted in cells by different regulatory factors (11, 15–25). Recent reports have called this work into question, suggesting that without specific buffering components, intrinsic chromatin condensates are solid, reflecting the globally constrained organization of chromatin in cells (26, 27). The distinction between liquid-like and solid-like behavior of chromatin condensates is important because many nuclear processes rely on dynamic rearrangements of chromatin. Whether such dynamics, especially those on short length scales, can occur through simple thermal fluctuations (as in a liquid-like state) or require input of energy (as in a solid-like state) impacts mechanistic considerations of many processes, and the ability of in vitro chromatin condensates to model them.

Here, we examine in detail the effect of solution conditions on the properties of intrinsic chromatin condensates. We find that condensates composed of small chromatin fragments are fluid, similar to a recent report (28); no unique solution composition is needed for their liquid-like properties. We also examine how sample preparation and imaging strategies can lead to mischaracterization of chromatin condensates. Last, we make efforts to clarify how the liquid-like organization of condensates might translate to chromatin dynamics in cells.

## Results

### Bovine Serum Albumin (BSA) and Dithiothreitol (DTT) Are Dispensable for the Liquid-Like Properties of Condensates Formed through Intrinsic Phase Separation of Chromatin.

In prior work (11), somewhat complex solutions were used to explore the nature of condensates formed from chromatin, most typically containing tris(hydroxymethyl)aminomethane (Tris) buffer, acetate, potassium, magnesium, BSA, DTT, ethylenediaminetetraacetic acid (EDTA), glycerol, and oxygen-scavenging components (glucose oxidase, catalase, and glucose). The composition of this solution was an effort to mimic the cellular milieu (acetate, potassium, BSA, glycerol, and DTT) and reduce photodamage of condensates during fluorescence microscopy (oxygen-scavenging components and DTT). Recent reports have suggested that BSA and DTT in these buffers lead chromatin condensates to exhibit artifactual liquid-like behavior and that their omission reveals the mesoscale material properties of condensates to be solid-like and constrained (26, 27). We set out to rigorously explore the effect of buffer conditions on chromatin condensate behavior.

**Fig. 1.**
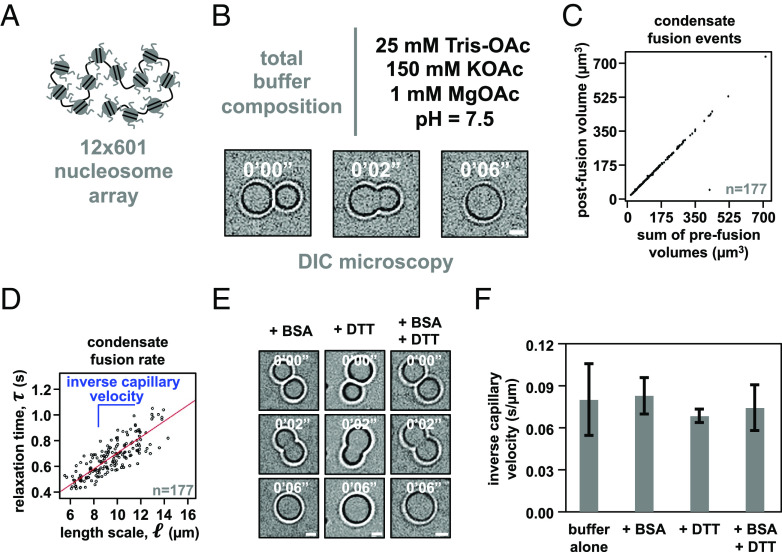
Intrinsic chromatin condensates are fluid without BSA and DTT. (*A*) Graphical depiction of the dodecameric nucleosomal arrays used for experimentation. (*B*) Differential interference contrast microscopy images of a fusion event between intrinsic chromatin condensates in the indicated buffer. (*C*) Dot plot representation of the inferred total volume of condensates before and after fusion. (*D*) Relaxation time versus length scale (sum of prefusion diameters) for 177 individual instances of condensate fusion in the buffer composition indicated in Fig. 1*B*. Inverse capillary velocity, the characteristic ratio of surface tension, η , and viscoscity, γ , is derived from the linear fit (red line) of the plots’ slope. (*E*) Differential interference contrast microscopy images of intrinsic chromatin condensate fusion in the buffer indicated in Fig. 1*B* supplemented with BSA (0.1 mg/mL, *Left*), DTT (5 mM, *Middle*), or BSA and DTT (0.1 mg/mL and 5 mM, respectively, *Right*). (*F*) Bar chart of inverse capillary velocities ( ± SD of 2 biological replicates) of intrinsic chromatin condensates in the buffer indicated in Fig. 1*B*, buffer with BSA, or buffer with BSA and DTT. For each condition, the fusion events per replicate are: buffer (177 and 68), +BSA (147 and 81), +DTT (183 and 68), +BSA+DTT (184 and 93). Scale bars, in white, are 4 μm.

We assembled dodecameric nucleosomal arrays by salt-mediated dialysis of reconstituted and unlabeled histone octamers and a DNA template with 12 repeats of Widom’s 601 nucleosome positioning element (Fig. 1*A*). Using differential interference contrast microscopy, we observed in a minimal phase separation buffer composed of 25 mM Tris-acetate, 150 mM potassium acetate, and 1 mM magnesium acetate the formation of micron-sized spherical condensates that rounded upon fusion (Fig. 1*B*) and maintained a consistent total volume following coalescence (Fig. 1*C*).

Droplet fusion followed by rounding to a spherical shape is a hallmark of fluids. The rate at which rounding occurs is a consequence of the relationship between the surface tension ( γ ) and viscosity ( η ) of condensates (29). Simple fluids coalesce according to the equation $\tau \approx \frac{\eta }{\gamma }\cdot l$ , where l is the diameter of condensates prior to fusion and τ is the characteristic relaxation time during coalescence. To determine τ for each instance of condensate fusion, we measured the change in aspect ratio ( AR ) over time ( t ) during condensate fusion and found these values fit well to an exponential decay, $AR=1+\left({\mathrm{AR}}_{\mathrm{init}}-1\right)\cdot e^{-t/\tau }$ , where ${\mathrm{AR}}_{\mathrm{init}}$ is the initial aspect ratio following the onset of fusion (*SI Appendix*, Fig. S1). Plotting τ versus l from many fusion events (*N* = 177) showed clear linearity, with relaxation times on the order of seconds, indicating that intrinsic chromatin condensates in this minimal buffer are fluid (Fig. 1*D*). The slope of this plot gives the inverse capillary velocity for these condensates in this solution, which is a quantitative measure of the distinctive ratio of surface tension ( γ ) and viscosity ( η ) of the material. We note that although this analysis reports on the viscosity of the solution, most biomolecular condensates are not simple Newtonian fluids but rather complex network fluids with viscoelastic behaviors. Viscoelasticity arises from the hierarchy of interaction strengths and timescales between the molecules (30–32). Full characterization of chromatin condensates will thus require rheological analyses across a range of length- and timescales, and is likely to reveal elasticity at small scales while viscosity dominates at the larger scales relevant to droplet fusion (and fluorescence recovery after photobleaching, FRAP, experiments below).

Condensates formed through intrinsic phase separation of chromatin in a solution containing BSA, DTT, or BSA and DTT also coalesced and became round (Fig. 1*E*). The inverse capillary velocity was identical within error for condensates formed in minimal phase separation buffer alone, or buffer with BSA, DTT, or BSA and DTT (Fig. 1*F*). These data show that BSA and DTT are not responsible for liquid-like material properties of intrinsic chromatin condensates.

### Condensates Formed by Intrinsic Phase Separation of Chromatin Are Liquid-Like in a Variety of Solutions.

We next explored how different anions and buffering systems affected the material properties of intrinsic chromatin condensates to ascertain whether their fluidity results from a particular component. We assayed the material properties of intrinsic chromatin condensates formed in solutions containing Tris buffer and sodium or potassium salts with chloride, acetate, or glutamate anions. Chloride is a typical anion used for biochemistry in buffered salt solutions. Previously, we used acetate to mimic small-molecule anions in cells; glutamate is the predominant anion found in cells (33). We also used piperazine-N,N′-bis(2-ethanesulfonic acid), pH adjusted with KOH (PIPES-KOH), a buffer/salt often used in fluorescence-based assays that reconstitute cellular processes, including microtubule dynamics (34, 35).

**Fig. 2.**
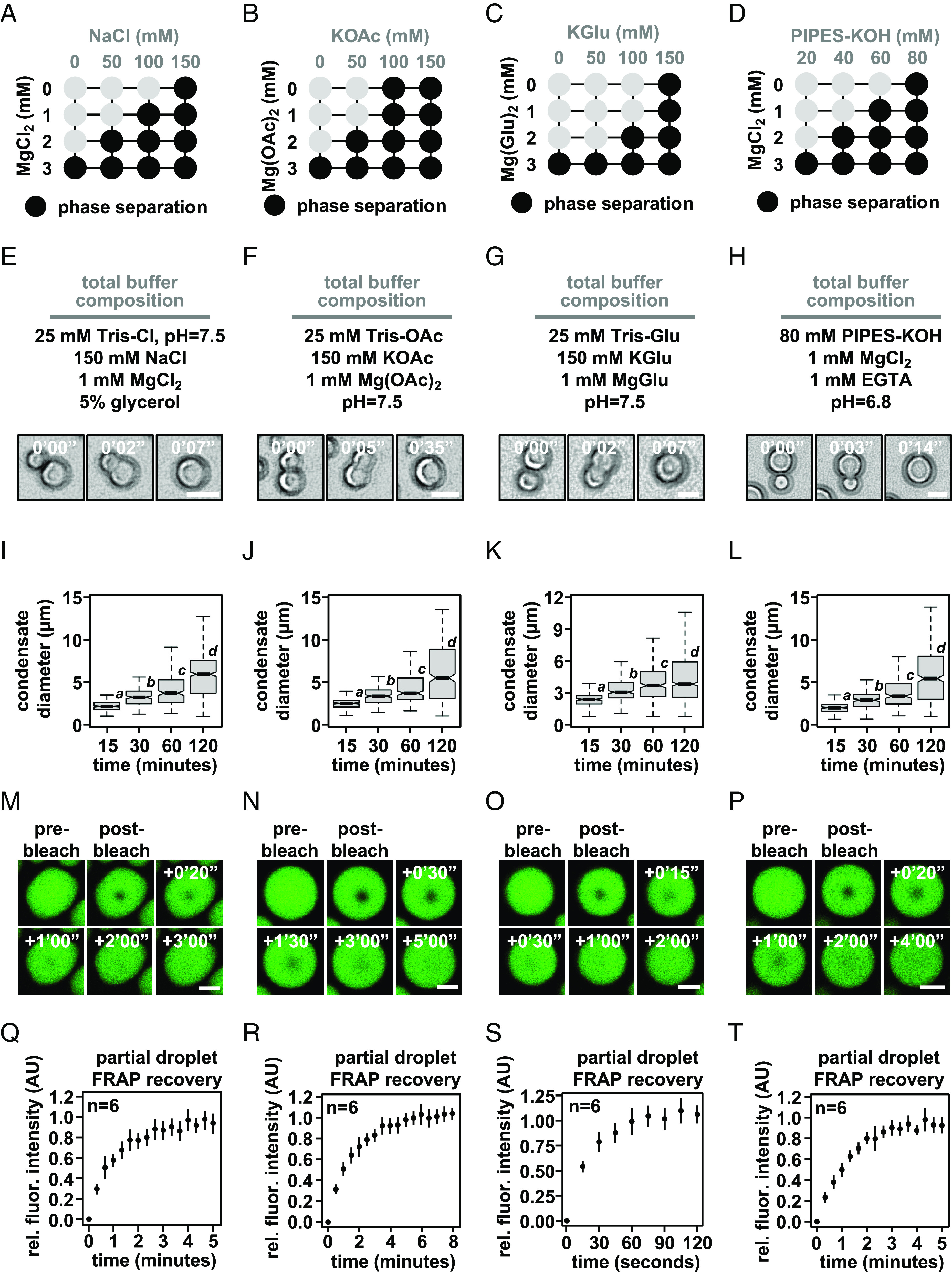
Intrinsic chromatin condensates are fluid in diverse buffers. Phase diagrams for intrinsic chromatin condensate formation in (*A*) Tris-chloride, (*B*) Tris-acetate, (*C*) Tris-glutamate, and (*D*) PIPES-KOH buffers. Dark circles indicate the presence of condensates, and representative images are in *SI Appendix*, Fig. S2. With materials produced and experiments performed in the Narlikar lab, bright-field light microscopy images of intrinsic chromatin condensate fusion in (*E*) Tris-chloride, (*F*) Tris-acetate, (*G*) Tris-glutamate, and (*H*) PIPES-KOH buffers. Boxplots of intrinsic chromatin condensate diameters following induction of phase separation in (*I*) Tris-chloride, (*J*) Tris-acetate, (*K*) Tris-glutamate, or (*L*) PIPES-KOH-based buffers. Bars marked with different letters are significantly different from one another (Student’s *t* test, *P* < 1 × 10^−7^). Fluorescence microscopy images of partial-droplet FRAP of intrinsic chromatin condensates, in green, composed of nucleosomal arrays labeled with AlexaFluor 488 in (*M*) Tris-chloride, (*N*) Tris-acetate, (*O*) Tris-glutamate, or (*P*) PIPES-KOH-based buffers. Quantification of partial-droplet FRAP of intrinsic chromatin condensates in (*Q*) Tris-chloride, (*R*) Tris-acetate, (*S*) Tris-glutamate, or (*T*) PIPES-KOH-based buffers. Fluorescence signal is normalized to pre-bleach droplet intensity and error bars are SD of six technical replicates. Scale bars, in white, are 4 μm.

First, we determined the phase diagram for dodecameric nucleosomal arrays at 500 nM nucleosome concentration for each buffer (Fig. 2 *A*–*D*). Condensates formed at similar concentrations of mono- and divalent salt in each buffering system, though glutamate anions required slightly higher concentrations of salt. In buffers containing chloride anion, condensate formation required at least 2 mM magnesium or the inclusion of glycerol (*SI Appendix*, Fig. S2 *A*–*F*). While the source of this effect is not clear, it could arise from the well-described propensity of glycerol to shield charged peptide side chains from salt (36). Altogether, these data show that intrinsic chromatin condensation occurs robustly across many buffer compositions.

For each buffering system, we chose a combination of mono- and divalent ions that resemble physiological concentrations in cells. In these solution conditions, both unlabeled (Fig. 2 *E*–*H*) and AlexaFluor 488-labeled nucleosomal arrays (*SI Appendix*, Fig. S2 *G*–*J*) rounded in seconds following fusion. Moreover, condensate size increased over the course of at least 2 h (Fig. 2 *I*–*L*), most likely through condensate fusion (11). These data suggest that in different buffers, intrinsic chromatin condensates are fluid.

To probe the dynamics of molecules within these condensates, we photobleached a portion of condensates and measured the recovery of fluorescence using condensates composed of AlexaFluor 488-labeled dodecameric nucleosomal arrays in each of the buffered salt solutions (Fig. 2 *M*–*T*). These partial-droplet fluorescence recovery after photobleaching (FRAP) experiments was carried out using glass treatments that reduce condensate motion (see below) to aid in the quantitation of photobleach recovery. This preparation affects condensates in chloride buffers more strongly than others, resulting in adherence to the surface and nonspherical shapes. Still, in each buffer condition, we observed rapid and full fluorescence recovery from photobleaching in minutes (Fig. 2 *Q*–*T*). Notably, condensates in buffers with glutamate, the predominate anion in cells, recovered approximately three-fold more rapidly from photobleaching as compared to chloride, acetate, and PIPES-KOH buffered salt solutions (based on t_1/2_ of fluorescence recovery). These data demonstrate that in a variety of simple buffers, intrinsic chromatin condensates are fluid.

### Condensate Fluidity Is Retarded by a Nonphysiologic Solution, but not by Several Other Factors.

The material properties of biomolecular condensates are an emergent phenomenon, where small differences between molecules and their interactions can impart substantial effects. We next sought to explore whether small differences in nucleosome arrays vs nucleosomal arrays or the conditions used to assay chromatin condensates might have significant effects on their dynamics and liquid-like behavior.

Reconstituting nucleosome arrays from bacterially purified components is a complex biochemical procedure (37), and small errors can result in underassembly, partial assembly, or overassembly of nucleosome arrays, which result, respectively, in free nucleosome positioning sequences, subnucleosomal structures (e.g., tetra- or hexasomes), or aggregates of nonnucleosomal histones on chromatinized DNA. Intrinsic chromatin condensates composed of improperly formed nucleosome arrays would likely affect their material properties, so we have accounted here for potential differences in the quality of nucleosome arrays by performing key experiments with independent materials from multiple laboratories with experience in chromatin reconstitution (Fig. 2 and *SI Appendix*, Fig. S2; also, see *Experimental Methods*), with each demonstrating clear liquid-like material properties. We first explored how long linker DNA lengths might affect chromatin droplet fluidity.

In cells, linker DNA length is highly regulated. While each eukaryotic organism, cell type, and genomic region can harbor short (~20 bp in *Saccharomyces cerevisiae*) to long (~90 bp in *Thyone briareus*) average linker lengths (38), across eukaryotes, there is a predisposition for nucleosomes to be placed every 10n+5 base pairs from one another (e.g., 5, 15, and 25) (39–41). 10n-spaced (e.g., 10, 20, and 30) polynucleosome arrays can adopt hierarchically folded two-start zig-zag fibers in vitro (42, 43), while 10n+5-spaced arrays prefer to interact with other chromatin fragments and form intrinsic chromatin condensates (11), demonstrating how the specific DNA template used in these assays can impact chromatin droplet formation and perhaps the material properties of the condensates that are formed.

**Fig. 3.**
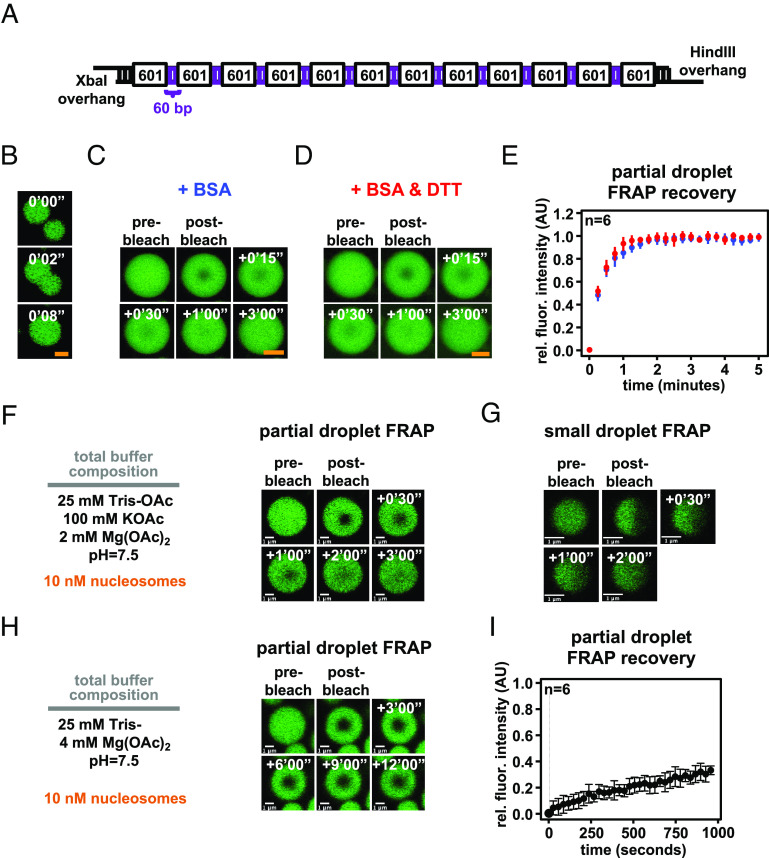
Intrinsic chromatin condensates are fluid in most conditions, but not in superphysiologic magnesium alone. (*A*) Graphical depiction of a long linker-length 12 × 601 DNA template (27). (*B*) Confocal fluorescence microscopy of intrinsic chromatin condensates composed of AlexaFluor 488-labeled long linker-length nucleosomal arrays, in green, undergoing fusion. Confocal fluorescence microscopy of partial-droplet FRAP of intrinsic chromatin condensates composed of AlexaFluor 488-labeled long linker-length nucleosomal arrays, in green, formed in the presence of (*C*) 0.1 mg/mL BSA or (*D*) 0.1 mg/mL BSA and 5 mM DTT. (*E*) Quantification of partial-droplet FRAP of intrinsic chromatin condensates with BSA or BSA and DTT, in blue and red, respectively. Fluorescence signal is normalized to pre-bleach droplet intensity and error bars are SD of six technical replicates. (*F*) Partial-droplet FRAP or (*G*) half-droplet FRAP of large or small intrinsic chromatin condensates, respectively, formed at 10 nM nucleosome concentration in minimal phase separation buffer. (*H*) Partial-droplet FRAP and (*I*) quantitation of fluorescence recovery for intrinsic chromatin condensates induced to form at 10 nM nucleosome concentration with 4 mM magnesium acetate. Scale bars, in orange and white, are 4 and 1 μm, respectively.

We assembled nucleosome arrays using a DNA template that purported to produce condensates with more solid-like material properties (27). This template has 60 bp internucleosome linker DNA lengths, longer than those we had previously employed (15 to 45 bp), and 4 bp palindromic single-stranded DNA overhangs, which might act as a source of nonnucleosomal valency for this template (Fig. 3*A*). We prepared chromatin using this DNA template and found that intrinsic chromatin condensates fused and rounded in seconds in a buffer lacking BSA or DTT, composed of 25 mM Tris-acetate, 150 mM potassium acetate, and 1 mM magnesium acetate (Fig. 3*B*). In partial-droplet FRAP assays in the presence of either BSA or BSA and DTT, these condensates each recovered in minutes within error of one another (Fig. 3 *C*–*E*). These experiments demonstrate that altered material properties do not arise from differences in DNA template or an effect from BSA in the presence of DTT.

We next tested whether the concentration of nucleosome arrays or size of chromatin condensates might alter their properties. We assembled chromatin condensates at 10 nM nucleosome concentration (0.83 nM nucleosome arrays) in a buffer composed of 25 mM Tris-acetate, 100 mM potassium acetate, and 2 mM magnesium acetate. In partial-droplet FRAP on large droplets and half-droplet FRAP on small droplets, recovery from photobleach occurred in minutes (Fig. 3 *F* and *G*), similar to condensates formed with 1 μM nucleosome concentrations (Fig. 2 *N* and *R*). These data demonstrate that chromatin concentration and condensate size do not appreciably change intrinsic chromatin condensate fluidity.

Last, we explored the dynamics of intrinsic chromatin condensates formed in superphysiologic concentrations of magnesium without monovalent salt. These, or similar, nonphysiologic conditions have sometimes been used to study chromatin self-assembly in the past (44). We formed chromatin condensates at 10 nM or 1 μM nucleosome concentration in a buffer composed of 25 mM Tris-acetate and 4 mM magnesium acetate and observed in each condition minimal recovery from photobleach in partial-droplet FRAP assays (Fig. 3 *H* and *I* and *SI Appendix*, Fig. S2*K*). These data demonstrate that without monovalent salt, chromatin condensates formed in 4 mM magnesium exhibit solid-like material properties. While low ionic strength in this buffer could lead to long lifetime charge–charge interactions, it is not clear why condensates in magnesium alone should have solid-like properties. Regardless of mechanism, networks of interaction between chromatin fragments likely differ within chromatin condensates formed with these nonphysiologic buffers, complicating interpretations regarding the behaviors of chromatin in cells.

### Sample Preparation Affects Condensate Movement and Internal Dynamics.

**Fig. 4.**
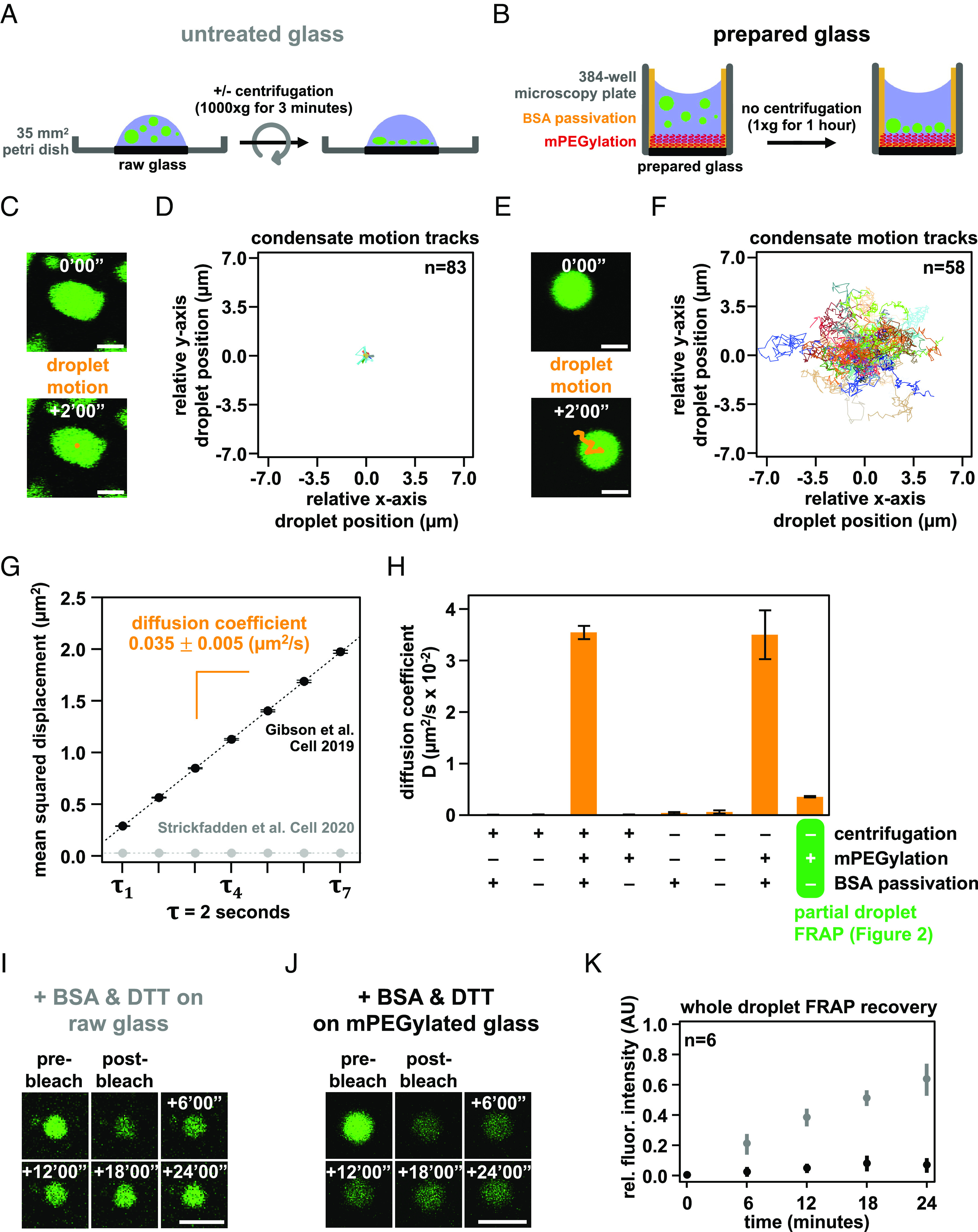
Condensate movement and dynamics is affected by microscopy glass preparation. Graphical depiction of techniques used to prepare intrinsic chromatin condensates for fluorescence microscopy imaging: (*A*) Intrinsic chromatin condensates can be spun onto raw glass using a centrifuge (27). (*B*) Alternatively, intrinsic chromatin condensates can be added to a 384-well microscopy plate and brought by gravity to rest on mPEGylated and BSA-passivated glass (11). Movement of a single or many intrinsic chromatin condensates, following their preparation for fluorescence microscopy imaging on untreated glass (*C* and *D*) and prepared glass (*E* and *F*). (*C* and *E*) The movement of an individual condensate across 2 min in 10 s intervals is overlaid in orange on fluorescence microscopy images of AlexaFluor 488-labeled intrinsic chromatin condensates, in green. (*D* and *F*) The relative movement of many condensates determined across 2 min in 500 ms intervals. (*G*) Plot of mean squared displacement ( ± SE) over lag time, τ , for intrinsic chromatin condensates between 4 and 8 μm in diameter following centrifugation onto untreated glass (gray dots) or settling by gravity onto prepared glass (black dots). The diffusion coefficient, indicated in orange ± SE, of intrinsic chromatin condensates can be calculated from the slope of the linear fit (dashed line) of the plotted data. For droplets centrifuged onto untreated glass, three replicates with 11,222, 8,114, and 14,092 trajectories extracted from 171, 147, and 238 droplets were used for analysis, respectively. For droplets settled onto passivated glass, three replicates with 6,563, 7,900, and 8,179 trajectories extracted from 106, 100, and 101 droplets were used for analysis, respectively. (*H*) Bar chart of the diffusion coefficients of intrinsic chromatin condensates following their preparation for microscopy with and without centrifugation, mPEGylation of the microscopy glass, and BSA passivation of the microscopy well. Error bars are SD of four technical replicates. Confocal fluorescence microscopy of whole-droplet FRAP of intrinsic chromatin condensates composed of AlexaFluor 488-labeled long linker-length nucleosomal arrays, in green, settled onto (*I*) untreated or (*J*) mPEGylated glass. (*K*) Quantification of whole-droplet FRAP recovery of intrinsic chromatin condensates on raw or mPEGylated glass, in gray and black, respectively. Fluorescence signal is normalized to pre-bleach droplet intensity and error bars are SD of six technical replicates. Panels *C*–*H* used nucleosome arrays with a 25 base pair internucleosome linker length. Panels *I*–*K* used nucleosome arrays with a 60 base pair internucleosome linker length. Scale bars, in white, are 4 μm.

Similar to single-molecule biochemical imaging studies (45), it is common when studying biomolecular condensates to prepare the cover glass surface to prevent artifactual wetting of biomolecules. In a study where intrinsic chromatin condensates were found to be solid-like (27), chromatin condensates were deposited onto raw glass by centrifugation prior to fluorescence microscopy (Fig. 4*A*). In our previous studies (11), we passivated the glass surface with methoxy polyethylene glycol (mPEG) and BSA to prevent the adherence of macromolecules and allowed condensates to settle onto the surface by gravity to minimize force-mediated perturbation (Fig. 4*B* and figure S1*E* of ref. 11). We investigated whether these differences affected the motion and physical properties of chromatin condensates.

Chromatin condensates deposited by centrifugation onto raw glass did not appreciably move during 2 min of observation by fluorescence microscopy and exhibited nonspherical morphology consistent with adhesion to the surface (Fig. 4 *C* and *D*). In contrast, intrinsic chromatin condensates settled by gravity onto mPEGylated and BSA-passivated glass moved many microns in distance, remained spherical, and underwent fusion (Fig. 4 *E* and *F*). We quantified the movement in these two conditions by measuring the mean squared displacement by lag time and found that condensates settled onto prepared glass were mobile, with a diffusion coefficient of 0.035 ± 0.005 μm^2^/s for condensates between 4 and 8 μm in diameter, while those deposited onto raw glass were not (Fig. 4*G* and *SI Appendix*, Fig. S3 *A*–*D* and *K*).

To understand what experimental parameter led to these differences, we quantified condensate movement with and without centrifugation, mPEGylation, and BSA passivation. Time-lapse imaging showed that diffusive condensate movement requires mPEGylation and BSA passivation, though some subdiffusive mobility is retained without passivation so long as glass is mPEGylated and condensates are not centrifuged onto the surface (Fig. 4*H* and *SI Appendix*, Fig. S3 *D*–*K*). The microscopy sample preparation can thus impact condensate movement and fusion.

We considered whether BSA leaching from the passivated glass surface might lead to liquid-like condensate properties. Three pieces of data argue against this possibility. First, our photobleaching experiments, which show rapid recovery, are carried out in the absence of BSA passivation (Fig. 2 *M*–*T*). Second, condensates move, albeit with restriction, in the absence of BSA passivation (Fig. 3*H*). Third, condensates fuse with comparable kinetics in the presence or absence of BSA passivation (*SI Appendix*, Fig. S3*L*). Thus, the liquid-like behavior of intrinsic chromatin condensates is not a consequence of BSA passivation.

Given the strong effects of slide surfaces on condensate movement, we next asked how glass treatment might affect the physical properties of the condensates themselves. Using long linker-length chromatin (Fig. 3*A*), we photobleached entire condensates to probe the extent of fluorescence recovery resulting from chromatin exchange between the condensed and dilute phases. This is distinct from partial-droplet FRAP in Fig. 3, which principally measures the movement of chromatin within a condensate. On raw glass, we observed appreciable recovery of fluorescence in the presence of BSA and DTT as described in other work (Fig. 4*I*) (27). In contrast, condensates settled onto mPEGylated glass did not substantially recover (Fig. 4 *J* and *K*), which we hypothesized previously (11) is due to the very low concentration of chromatin in solution (note that differences in partial versus whole-droplet FRAP recoveries were addressed in our previous study). These data show that microscopy preparations affect not just the movement of intrinsic chromatin condensates, but also their exchange with molecules in solution. While we do not understand the basis of this difference, condensates centrifuged or settled to a strongly adherent glass surface will be flattened, perhaps appreciably so. In contrast, condensates settled onto a well-passivated surface will remain spherical and shielded from the glass. The additional interactions between a flat condensate and glass may influence photobleaching recovery and might exhibit sensitivity to specific buffering components.

### BSA and DTT Mitigate Photocrosslinking of Intrinsic Chromatin Condensates.

Having analyzed how differences in sample preparation can alter condensate movement and FRAP recovery, we next examined the effects of imaging parameters. Laser excitation can produce radical oxygen species (ROS) that react with and crosslink neighboring molecules. Such light-induced crosslinking can cause artifactual hardening of biomolecular condensates (46). ROS production and photocrosslinking of molecules are typically mitigated in biochemical imaging studies by including reducing agents in buffers, limiting fluorophore concentration, minimizing laser excitation, and scavenging soluble oxygen in solution (47–49). In a previous report where biochemical experiments were performed without these additions (27), intrinsic chromatin condensates demonstrated solid-like behavior, raising the possibility that photocrosslinking might have limited chromatin mobility in their condensate imaging experiments. We therefore explored the effect of ROS mitigation on photocrosslinking of intrinsic chromatin condensates.

**Fig. 5.**
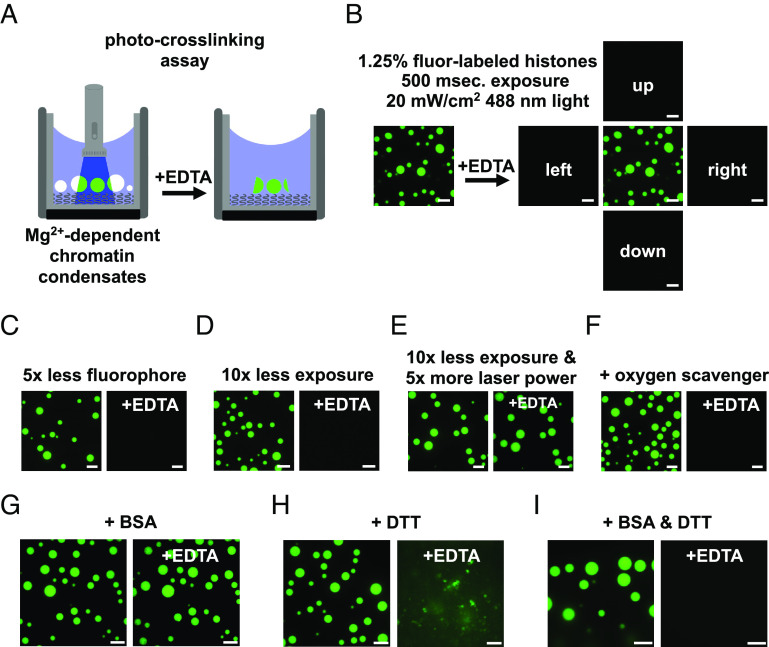
DTT and BSA mitigate photocrosslinking during fluorescence microscopy. (*A*) Diagram depicting an assay to detect photocrosslinking of intrinsic chromatin condensates. (*Left*) Magnesium-dependent intrinsic chromatin condensates are exposed to fluorescent light prior to the addition of superstoichiometric quantities of EDTA. (*Right*) Photocrosslinked condensates fail to dissipate following chelation of magnesium. (*B*) Confocal fluorescence microscopy images of intrinsic chromatin condensates composed of nucleosomal arrays where 1 in 80 histone molecules are labeled with AlexaFluor 488. Images are following exposure to fluorescent light and both before (*Left*) and after (*Right*) the addition of EDTA. Confocal fluorescence microscopy images of intrinsic chromatin condensates imaged, as in Fig. 5*B*, with (*C*) less fluorophore, (*D*) less exposure, (*E*) more laser power with less exposure, or (*F*) the inclusion of oxygen scavenging components. Confocal fluorescence microscopy images of intrinsic chromatin condensates formed in the presence of (*G*) BSA, (*H*) DTT, or (*I*) BSA and DTT and imaged as described in Fig. 5*B*. Fluorescent microscopy images before and after the addition of EDTA were processed separately. All experiments were performed using nucleosome arrays with 25 base pair internucleosome repeat length. Scale bars, in white, are 10 μm.

We developed an assay to measure light-induced photocrosslinking of intrinsic chromatin condensates. In this assay, condensates were formed in a buffer where free magnesium was required for their formation (Fig. 2*B*, 2 mM Mg (OAc)_2_ and 50 mM KOAc). The concentration of monovalent salt in this buffer is insufficient to induce nucleosomal arrays to phase separate. Under these conditions, condensates can be dissolved by chelation of magnesium with EDTA (Fig. 5*A*). We hypothesized that photocrosslinking condensates would prevent their dissolution by EDTA.

We formed intrinsic chromatin condensates with 1 in 80 histone proteins conjugated to a fluorophore in a magnesium-dependent phase separation buffer. Exposure of these condensates to 20 W/cm^2^ of fluorescent light for 500 ms ( λ= 488 nm), comparable to that used on our microscope in a typical imaging experiment, prevented their dissolution by EDTA (Fig. 5*B*). Condensates in adjacent fields, which had not been exposed to light, were dissolved 1 min after the addition of EDTA. Light-induced solidification of condensates did not occur with fivefold less fluorophore or 10-fold less light (Fig. 5 *C* and *D*). Shorter exposure to light of higher intensity also led to condensate solidification, demonstrating that the totality and not duration of light exposure drives condensate solidification (Fig. 5*E*). Addition of an oxygen scavenging system to the buffer prevents light-induced condensate solidification (Fig. 5*F*), although its inclusion can alter condensate properties (*SI Appendix*, Fig. S4). Together, these data demonstrate that imaging intrinsic chromatin condensates can cause their solidification and suggest that this results from light-induced ROS production and photocrosslinking. Furthermore, these data highlight how minimizing light exposure, fluorophore density, and including oxygen scavengers can prevent artifactual hardening of condensates.

We next sought to understand how the inclusion of BSA and/or DTT can influence photocrosslinking of intrinsic chromatin condensates. Adding 100 ng/μL BSA, as used in our own and other studies (11, 26, 27), did not prevent condensate solidification (Fig. 5*G*). In 5 mM DTT, light exposure and EDTA addition resulted in loss of spherical condensates but left aggregates in solution, suggesting partial but incomplete mitigation of photocrosslinking (Fig. 5*H*). Adding BSA and DTT together prevented condensate solidification, enabling their dissolution upon EDTA addition. While the mechanism by which BSA, or some component in commercially available BSA, can inhibit photocrosslinking is unclear, these observations suggest that BSA and DTT can act in concert to reduce light-induced hardening of intrinsic chromatin condensates (Fig. 5*I*).

### Intrinsic Chromatin Condensates Show Length-Dependent Dynamics.

**Fig. 6.**
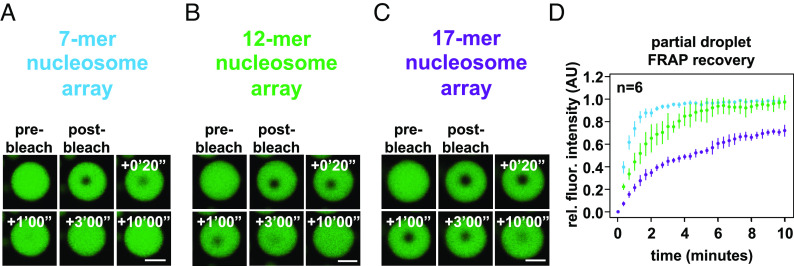
Length-dependent effects on chromatin condensate dynamics. Confocal fluorescence microscopy images of partial-droplet FRAP of intrinsic chromatin condensates, in green, composed of AlexaFluor 488-labeled arrays that are (*A*) 7, (*B*) 12, or (*C*) 17 nucleosomes in length. (*D*) Quantification of partial-droplet FRAP of intrinsic chromatin condensates composed of 7, 12, or 17 nucleosome-long arrays in blue, green, and purple, respectively. Fluorescence signal is normalized to pre-bleach droplet intensity and error bars are SD of six technical replicates. Scale bars, in white, are 4 μm.

The cellular chromatin polymer is vastly longer than the nucleosome arrays investigated here. According to classical polymer theory, this additional length would add constraints on polymer movement due to increased adhesion to neighboring molecules (50). As a step toward addressing this issue, we reconstituted chromatin in vitro with 7, 12, or 17 nucleosomes by altering the number of repeats of Widom’s 601 nucleosome positioning sequence, while keeping the internucleosome linker lengths constant. Chromatin condensates composed of these arrays were formed at 1 μM nucleosome concentration in a physiologic salt solution and assayed for changes in their dynamics using FRAP (Fig. 6 *A*–*D*). We found that increased chromatin length results in more limited recovery from photobleach. Condensates composed of even longer nucleosome arrays would be expected to exhibit more solid-like properties, as demonstrated recently with other biomolecular condensates (51, 52). Still, for very long polymers, short sections will retain dynamics at short length scales while moving little at longer lengths (50). Thus, an intrinsic chromatin condensate composed of chromosome-length fragments would be locally dynamic but exhibit little recovery from photobleach, like the dynamics of the genome observed in cells (53).

## Discussion

### The Liquid-Like Properties of Intrinsic Chromatin Condensates.

Here, we present data demonstrating that intrinsic chromatin condensates composed of short nucleosome arrays are fluid (likely viscoelastic) over the course of minutes (in FRAP and droplet fusion assays) under a wide range of physiologically relevant solution conditions. Quantification of rounding after fusion and partial-droplet FRAP recovery show that BSA and DTT impart no effect on condensate fluidity, even when using a DNA template that had exhibited solid-like behaviors (27). Others have recently come to similar conclusions (28). From a series of experiments, we show that fluid condensates can appear solid-like without passivation of glass or when ROS-limiting components are omitted. Our results have important implications on the behavior of chromatin and the use of phase-separated chromatin condensates to study nuclear processes.

### Regulated Solidification of Chromatin Assemblies in Cells.

We have shown intrinsic chromatin condensates are fluid, but it remains possible that chromatin assemblies may solidify in cells even on short length scales as part of a regulated biological process. ROS can crosslink and solidify chromatin (Fig. 5) and are produced in cells as a by-product of cellular processes. ROS are produced at large by mitochondrial metabolism or inflammatory cell signaling (54), and at specific genomic loci by enzymes like lysine-specific demethylase 1, whose removal of histone lysine methylation produces not just hydrogen peroxide, but also formaldehyde, which can crosslink and arrest chromatin movement (6, 55). It will be interesting to examine in future studies whether, and how, chromatin dynamics might be slowed to a solid-like state as part of normal cellular signaling and functions.

### Bridging Fluid Condensates to Chromatin Dynamics in the Cell.

A large body of data on the spatial organization and movement of loci in different cell types has demonstrated that on short length scales chromatin is highly dynamic. Superresolution and single-molecule fluorescence imaging have shown nucleosomes compact into 30 to 50 nm chromatin assemblies called “clutches” (56), which further assemble into chromatin domains with a radius of ~100 to 300 nm (57–61). Analyses of their motion have shown that individual nucleosomes move within these domains on tens of milliseconds timescales (62, 63) and the domains themselves move on hundreds of milliseconds to seconds timescales (6, 57, 58, 61, 64, 65). In both regimes, movement is subdiffusive and/or confined (6, 58, 61, 63–66), in part due to constraints on a given chromatin segment imparted by adhesions to surrounding structures, which increase with length of the segment (i.e., number of adhesions) (64, 67).

While poorly understood ATP-dependent processes can affect longer-length chromatin motion (65, 68), movement at small scales (e.g., short chromatin assemblies, limited radius) is thought to primarily occur via passive thermal fluctuations rather than actively driven processes (6, 57, 58, 64, 65, 67). Thus, short range/timescale movement reflects the dynamics of local internucleosome contacts that are subject to changes induced by histone acetylation and binding of linker histone H1 (59, 63). These local dynamics are likely necessary for many genome functions, such as enhancer–promoter interactions (69), loop extrusion by SMC complexes (70, 71), and homologous pairing of sequences during meiosis and DNA repair (71, 72). Lack of movement at greater scales (~400 nm or larger) arises from multiple constraints, including the large size of chromosomes, crosslinking macromolecules (e.g., SMC complexes, adaptor proteins), and attachment of chromatin to nuclear structures (e.g., nuclear bodies, nuclear lamina) (6, 66, 73–75). These constraints lead to the well-described reticence of chromatin in cells to recover from photobleaching (27, 73, 76–80). In condensates that form through interactions between small chromatin fragments alone, these larger-scale constraints are not present, allowing micrometer-scale movement and photobleach recovery. These long-range behaviors of intrinsic chromatin condensates in vitro very likely reflect the interactions that govern short length/timescale chromatin dynamics in cells (81). As numerous cellular processes depend on short-range chromatin dynamics, the reported absence of dynamics in chromatin condensates in vitro (26, 27) is thus unlikely to be physiologic, except perhaps in very specific biological situations (see above).

The length-dependent FRAP recovery behaviors shown in Fig. 6 underscore an important issue when studying condensates in vitro. Decades of study have demonstrated that the structure and function of discrete macromolecular complexes in vitro inform in a straightforward fashion on the structure and function of those factors in vivo. In contrast, the properties of condensates generated in vitro (e.g., size, structure, and behavior) require care in their translation to cellular correlates. In this regard, we propose that factors that influence “mesoscale” genome dynamics in cells will not be readily observable when studying intrinsic chromatin condensates generated from kilobase-scale DNA stretches. Mesoscale genome dynamics, defined as the larger-scale motion that determines photobleach recovery of chromatin in cells, are likely governed by short-range chromatin interactions translated to genome-relevant scales in the context of complicating factors that crosslink and adhere chromatin to physical structures of the nucleus. The utility of the reconstituted system of phase-separated nucleosomal arrays is the ability to study how factors influence short-range chromatin dynamics using a macroscopic technique like FRAP.

## Experimental Methods

Detailed methods for expression and purification of recombinant proteins and DNA, assembly of nucleosome arrays, preparation of slide surfaces, imaging, condensate crosslinking and fusion assays, and image analysis are provided in *SI Appendix*.

## Lead Contact and Materials Availability

Further information and requests for resources and reagents should be directed to and will be fulfilled by the Lead Contact, Michael K. Rosen (michael.rosen@utsouthwestern.edu).

## Supplementary Material

Appendix 01 (PDF)

## Data Availability

Datasets and software are available by requests to the corresponding author. Microscopy images data have been deposited in Dryad (doi:10.5061/dryad.83bk3j9ws) (82).
