# Supplementary material for: In diverse conditions, intrinsic chromatin condensates have liquid-like material properties
Source: Proc Natl Acad Sci U S A. 2023 Apr 24;120(18):e2218085120. doi: 10.1073/pnas.2218085120 (PMC10161002; doi:10.1073/pnas.2218085120)
Supplement: Supplementary file 1 — Appendix 01 (PDF) [file pnas.2218085120.sapp.pdf]

Supporting Information for

In Diverse Conditions Intrinsic Chromatin Condensates have Liquid-like Material Properties

Bryan A. Gibson, Claudia Blaukopf, Tracy Lou, Lifeng Chen, Lynda K. Doolittle, Ilya Finkelstein, Geeta J. Narlikar, Daniel W. Gerlich, Michael K. Rosen

Corresponding author: Michael K. Rosen
Email: michael.rosen@utsouthwestern.edu

This PDF file includes:

Supporting text
Figures S1 to S4
SI References

Supporting Information Text

Bacterial Strains. DH5α (Invitrogen) and MACH1 (Invitrogen) *E. coli* strains were used for passage during cloning of plasmid DNA. Large-scale preparations of plasmid DNA for isolation of nucleosome assembly sequences were passed through and grown to scale in the ER2925 (*dam⁻/dcm⁻*) *E. coli* strain (NEB). Histone proteins were expressed in Rosetta2(pLysS) E. coli cells (Novagen) in the Rosen lab and in BL21(DE3) pLysS cells (Agilent) in the Narlikar lab.

Molecular Biology and Cloning.

Construction of 12x601 dsDNA Array-Producing Bacterial Vectors.

193bp Repeat Length TetO-containing 12x601: The p12x601 insert from Gibson et al Cell 2019 was subcloned into the WM530 plasmid (a generous gift from Tom Muir) to create pWM+12x601 plasmid. This plasmid contains a 12x601 array with Tet Operator (TetO) inserted between 601 sequences 6 and 7 with 46 bp DNA lengths between nucleosome positioning sequences. Digesting this plasmid with EcoRV fragments the DNA backbone into < 500 bp sizes (e.g., carrier DNA) and liberation of the 12x601 array.

7x601 and 17x601 Nucleosomal Arrays: pUC19 with a 7x601 insert containing 25 base pair DNA segments between nucleosome positioning sequences was subcloned from pUC19+6x601_25bplinker (1) and pUC19+3x601_25bplinker plasmids (1) to create pUC19+7x601_25bplinker. pUC19 with a 17x601 insert containing 25 base pair DNA segments between

nucleosome positioning sequences was subcloned from pUC19+7x601_25bplinker and pUC19+12x601_25bplinker (1) plasmids to create pUC19+17x601_25bplinker. Of note: 2 601 repeats are lost during isoschizomer digestion and ligation when subcloning these 601-repeat containing constructs.

601 array DNA within pUC19+7x601_25bplinker and pUC19+17x601_25bplinker were subcloned into WM530 to create pWM+7x601 and pWM+17x601. Digesting this plasmid with EcoRV fragments the DNA backbone into < 500 bp sizes (e.g., carrier DNA) and liberation of the 12x601 array.

Expression and Purification of Recombinant Proteins in the Rosen Lab.

Purification of H. sapiens Histone Proteins Expressed in E. coli.

Expression: Histones were expressed exactly as previously described (1). Briefly, pET-based plasmids encoding human histone proteins ($H3_{C111A}$, H4 , H2A.1, H2B, or H2BT116C) were transformed into Rosetta2(pLysS) E. coli cells (Novagen) and grown to a density (OD600) of 0.4 at 37°C. Recombinant protein expression was induced by addition of IPTG to 1 mM for 3 hours at 37°C. The cells were collected by centrifugation, resuspended in Histone Lysis Buffer (50 mM Tris•HCl, pH 8, 150 mM NaCl, 5 mM ß-mercaptoethanol, 1 mM Benzamidine, 100 µM Leupeptin, 100 µM Antipain, 1 µM Pepstatin), flash frozen in liquid N_2, and stored at -80°C.

Purification: Histones were purified essentially as previously described (1). *E. coli* expressing histone proteins were passed through an Avestin Emulsiflex-C5 high pressure homogenizer at ~10,000 PSI. Inclusion bodies were washed twice

by centrifugation and resuspension with Inclusion Body Wash Buffer (50 mM Tris•HCl, pH 7.5, 100 mM NaCl, 1% Triton X-100, 1 mM EDTA, 1 mM Benzamidine, 5 mM ß-mercaptoethanol) and twice more omitting Triton X-100. Inclusion Bodies were soaked with DMSO, minced with a spatula, extracted with Histone Unfolding Buffer (20 mM Tris•HCl, pH 7.5, 7M Guanidinium-HCl, 10 mM DTT). Extracted unfolded proteins centrifuged, filtered through a 0.45 µm membrane (GE Healthcare) and run in Histone Unfolding Buffer over a HiLoad 26/60 Superdex 200 pg size exclusion column. Fractions containing histone proteins were dialyzed three times against 5 mM ß-mercaptoethanol. Any precipitate was pelleted by centrifugation.

Soluble histone protein was reduced in volume in a centrifugal concentrator before dilution with > 20 volumes of SAU200 (20 mM NaOAc, pH 5.2, 7 M Urea, 200 mM NaCl, 1 mM EDTA, 5 mM ß-mercaptoethanol). Histone proteins in SAU200 were filtered and purified by cation exchange chromatography (Source 15S) using SAU200 (20 mM NaOAc, pH 5.2, 7 M Urea, 600 mM NaCl, 1 mM EDTA, 5 mM ß-mercaptoethanol) as an eluate. Fractions containing histone proteins were dialyzed three times against 5 mM ß-mercaptoethanol, reduced in volume in a centrifugal concentrator, quantified by measuring solution absorbance at 280 nm and using the calculated molar extinction coefficients (https://web.expasy.org/protparam/) for histones H3C111A, H4, and H2A of 4470/M•cm, 5960/M•cm, and 4470/M•cm, respectively. Purified histone proteins were aliquoted in single use quantities, flash frozen with liquid N_2, and stored at -80 ˚C.

Labeling Histone H2BT116C with AlexaFluor 488

Histone H2B mutant H2BT116C was labeled exactly as previously described (1). Briefly, cysteines reduced by adding TCEP to 1 mM final concentration at room temperature for 1 hour. Following salt exchange of Histone H2BT116C into Phosphate Buffered Saline (8 mM Na_2HPO_4, 2 mM KH_2PO_4, pH 7.4, 137 mM NaCl, 2.7 mM KCl) using HiTrap Desalting Columns (GE Healthcare) 1.5 molar excess Alexa Fluor 488 (AF488)-C_5-maleimide was added. Following a 4-hour incubation in the dark, DTT was added to 10 mM final concentration to quench the reaction. Free fluorophore was removed by passing fluor-conjugated histone H2B a desalting column and anion exchange chromatography (Source 15Q) in Histone CleanUp Buffer (20 mM Tris•HCl, pH 7.5, 150 mM NaCl, 1 mM DTT). Fractions containing AF488-labeled histone H2B proteins were dialyzed three times against 5 mM ß-mercaptoethanol and reduced in volume using centrifugal concentrators. Protein concentration and percent labeling were quantified by measuring absorbance at 280 and 495 nm and the calculated molar extinction coefficients (https://web.expasy.org/protparam/ or Thermo Scientific) for histone H2BT116C and AlexaFluor488 of 4470/M•cm and 73000/M•cm, respectively. 100% labeling was confirmed and AlexaFluor 488-labeled histone H2B protein was aliquoted for single use, flash frozen with liquid N_2, and stored at -80˚C.

Expression and Purification of Recombinant Proteins in the Narlikar Lab. *Xenopus laevis* histones were expressed in BL21(DE3) pLysS cells (Agilent) and purified from *E.coli* as previously described (2).

Reconstitution of Histone Octamers in the Rosen Lab. Histone Octamers were reconstituted essentially as previously described (1). Briefly, aliquots of histone proteins were mixed in Histone Unfolding Buffer at a final concentration of 16.7:16.7:20:20 nmol per mL for H4:H3:H2B:H2A. The histone mix was dialyzed three times against Refolding Buffer (10 mM Tris•HCl, pH 7.5, 2 M NaCl, 1 mM EDTA, 5 mM ß-mercaptoethanol). The dialysate was filtered by passage through a 0.45 µm filter and reduced in volume using a centrifugal concentrator. Refolded histone octamer was isolated by size exclusion chromatography on a HiLoad SD200 26/60 pg column. Peak Fractions were analyzed by SDS-PAGE analysis for equal stoichiometry of core histone proteins, pooled, and reduced in volume with a centrifugal concentrator. The absorbance of reconstituted histone octamers was measured at 280 nm, (and 495 nm for fluorophore-labeled octamers) and concentration of protein was calculated using the molar extinction coefficients (https://web.expasy.org/protparam/ or Thermo Scientific) for histone octamer and AF488 of 44700/M•cm and 73000/M•cm, respectively. 100% labeled histone octamers were confirmed by the presence of 2:1 stoichiometric excess of fluor to histone octamer. Purified histone octamers were aliquoted, flash frozen with liquid N_2, and stored at -80 ˚C. Note: The final concentration of histone octamers used

for chromatin assembly was adjusted by their capacity to assemble single 601 sequences into mononucleosomes.

Reconstitution of Histone Octamers in the Narlikar Lab. Histones were refolded in high salt buffer to form octamer and purified by size-exclusion chromatography as previously described (2).

Preparation of DNA templates for Chromatin Assembly in the Rosen Lab. Plasmids containing 601 repeat DNA were prepared largely as previously described (1). Briefly, plasmids containing 601 repeat DNA were transformed into dam$^-$/dcm$^-$ *E. coli* strain ER2925 and plated onto LB agar plates supplemented with 100 ng/µL Ampicillin for growth overnight. Following small scale growth to turbidity from a single colony, 4.5 liters of LB with 100 ng/µL of Ampicillin were inoculated with bacteria for overnight growth at 37 ˚C. Bacteria was harvested by centrifugation and plasmid DNA was purified using a Qiagen Plasmid Giga Kit. 601 array DNA was liberated from the plasmid backbone and backbone DNA digested to small fragments (e.g., carrier DNA) using restriction endonuclease digestion. pWM+12x601, pWM+12x601_25bplinker, and pWM+17x601_25bplinker were digested with the EcoRV-HF restriction endonuclease. pWM+7x601_25bplinker was digested with EcoRV-HF, MSPA1I, TaqI-V2, and AvaII restriction endonucleases. p12x207, used in Strickfaden et al. Cell 2020 (A gift from Jeffrey Hansen), was digested with XbaI, HindIII, MspA1I, AatII, BsaWI, DraI, and BglI. Following backbone digestion and 601 array DNA

liberation, DNA was purified by Phenol:Chloroform:Isoamyl Alcohol (25:24:1) Extraction and Ethanol Precipitation. DNA was resuspended for storage in 1xTE (10 mM Tris•HCl, pH 7.5, 1 mM EDTA) and quantified using a Nanodrop (ThermoFisher Scientific).

Preparation of DNA templates for Chromatin Assembly in the Narlikar Lab. The array DNA template was isolated by restriction enzyme digest of plasmid containing 12x601s (1). Array DNA was purified using the Gigaprep Kit (Qiagen) and by size-exclusion. This was followed by ethanol precipitation and resuspension in 1X TE.

Preparation of Nucleosomal Arrays in the Rosen Lab.

Setup of Nucleosomal Assemblies. Nucleosomes were setup for assembly largely as previously described (1). Briefly, DNA and histone octamers were thawed on wet ice. An equal volume of 4M Assembly Buffer (10 mM Tris•HCl, pH 7.5, 1 mM EDTA, 4 M NaCl, 2 mM DTT) was added to DNA (in 1xTE). Histone octamers were added in slight stoichiometric excess relative to 601 nucleosome positioning sequences in the template (1.3:1), to ensure full assembly in the presence of carrier DNA which prevents overassembly. Final concentrations of octamer/601 was 5 µM. Histone octamers and 601-containing DNA templates were moved into dialysis chambers equilibrated in High Salt Assembly Buffer (10 mM Tris•HCl, pH 7.5, 1 mM EDTA, 2 M KCl, 1 mM DTT).

Salt Dialysis-mediated Assembly of Nucleosomes. Nucleosomes were assembled as previously described (1). The salt concentration in dialysis chambers containing histones and DNA were lowered over three days by continuous dilution using Low Salt Assembly Buffer (10 mM Tris•HCl, pH 7.5, 1 mM EDTA, 200 mM KCl, 1 mM DTT) at 4˚C. After three days dialysis chambers were dialyzed against 1xTE with 1 mM DTT for at least for hours at 4˚C.

Sucrose Gradient-mediated Purification of Nucleosomal Arrays. Following salt-mediated dialysis, assembled nucleosomal arrays were applied to linear 15-40% (12- or 17-nucleosome arrays) or 5-25% (7-nucleosome arrays) sucrose gradients in 1xTE with 1 mM DTT. Sucrose gradient fractions containing assembled nucleosomal arrays were dialyzed into 1xTE with 1 mM DTT and concentrated in using centrifugal concentrators.

Quantitation of Nucleosome Concentration. To quantitate final chromatin concentrations, 10 µL of nucleosomal arrays were added to 90 µL of SDS/PK Buffer (45 mM Tris•HCl, pH 7.5, 9 mM EDTA, 1% SDS) and incubated for 30 minutes at room temperature. DNA was purified using a Qiagen PCR purification Kit and quantified using a Nanodrop spectrophotometer.

Quality Assurance of Nucleosomal Assembly. The quality of nucleosome assemblies was assessed for by digesting linker DNA between 601 sequences in template DNA and chromatin using a restriction endonuclease for 1 hour at room temperature and running the digests on a native PAGE gel in 0.5X TAE. Only chromatin without unassembled 601 sequences, a clear shifted

mononucleosome band, and at most a trace hexasome population were used for experimentation.

Preparation of Nucleosomal Arrays in the Narlikar Lab. 12-mer nucleosome arrays were generated from a salt gradient dialysis to assemble histone octamer onto the 12x601 DNA as previously described (3). After assembly, arrays were dialyzed into TCS0.1 (20mM HEPES pH7.5, 0.1mM EDTA, 2mM 2-Mercaptoethanol).

Preparation of 384-well Microscopy Plates in the Rosen Lab.

mPEGylation of Silica. 384-well microscopy plates (Brooks Life Science Systems Matriplate) were washed with 5% Hellmanex at 37˚C for 4 hours and then rinsed copiously with ≥ 18 MΩ H_2O. Silica was etched with 1 M NaOH for 1 hour at room temperature and then rinsed copiously with ≥ 18 MΩ H_2O. Depolymerized Silica was covalently bonded overnight (≥ 18 hours) at room temperature to 20 mg/mL 5K mPEG-silane (PEGWorks) suspended in 95% Ethanol. Plate was washed many times with 95% Ethanol, rinsed with copious amounts of ≥ 18 MΩ H_2O, and completely dried in a chemical hood over 3-4 hours. PEGylated microscopy plate was sealed until individual wells' use with an adhesive PCR plate foil (Thermo).

Passivation of Well with Bovine Serum Albumin. Following PEGylation, foil was cut above individual wells prior to their use and both plastic and PEGylated glass were passivated by incubation with freshly prepared 100 mg/mL BSA for 30

minutes to 4 hours. Wells were rinsed three times with ≥ 18 MΩ H_2O to remove BSA and microscopy samples (15-40 µL in volume) were immediately added to the empty well. Desiccation of microscopy samples was limited following their addition to the plate by sealing with transparent scotch tape.

Preparation of 384-well Microscopy Plates in the Narlikar Lab.

mPEGylation of Silica. Wells in a microscopy plate were washed twice with ddH_2O before adding 2% Hellmanex. After a 1-hour incubation at room temperature the Hellmanex solution was removed, and wells were washed three times with ddH_2O. 0.5 M NaOH was added to the wells to etch the glass for 30 minutes at room temperature. The NaOH solution was removed and wells were washed three times with ddH_2O before adding 20 mg/mL mPEG-silane dissolved in 95% Ethanol. Wells were covered with foil tape and left to sit overnight in the dark. Nearly all of the mPEG-silane was removed before 10 serial washes with 95% EtOH followed by three washes with ddH_2O. Through each wash trace amounts of liquid were retained in the well to prevent desiccating the PEG layer conjugated to glass.

Passivation of Well with Bovine Serum Albumin. Following PEGylation, 100 mg/mL BSA was added to passivate the well for 2 hours at room temperature. Wells were rinsed three times with ddH_2O, finally leaving water in the well to await transfer of phase-separated chromatin.

Preparation of 384-well Microscopy Plates in the Gerlich Lab.

mPEGylation of Silica. 384-well µClear® microscopy plates (Greiner Bio-One, 781906) were washed with 5% Hellmanex in ≥ 18 MΩ H_2O at 65˚C for 4 hours in a tabletop Incu-Line oven (VWR) then rinsed 10 times with ≥ 18 MΩ H_2O. Silica was etched with 1 M KOH for 1 hour at room temperature and then rinsed 10 times with ≥ 18 MΩ H_2O. The etched multi-well plate was treated with 5K-mPEG-silane (Creative PEGWorks, PLS-2011) suspended in 95% Ethanol (VWR) for 18 hours at room temperature. The plate was washed once with 95% Ethanol, then 10 times with ≥ 18 MΩ H_2O, before completely drying in clean chemical hood overnight. Until their use, wells were sealed using an adhesive PCR foil (Thermo Fisher Scientific) and kept in a dry dark space.

Passivation of Well with Bovine Serum Albumin. Following PEGylation, foil was cut above individual wells prior to their use and both plastic and PEGylated glass were passivated by incubation with freshly prepared 100 mg/mL BSA for 30 minutes to 4 hours. Wells were rinsed three times with ≥ 18 MΩ H_2O to remove BSA and microscopy samples were immediately added to the empty well. Desiccation of microscopy samples was limited following their addition to the plate by sealing with transparent scotch tape.

Imaging Condensates in the Rosen Lab.

Phase Separation of Nucleosomal Arrays. Nucleosomal arrays with a 25 bp internucleosome linker DNA length and 1 in 100 fluorophore-labeled histone H2B proteins (unless otherwise indicated) were diluted in a minimal chromatin dilution buffer. For Tris-chloride conditions with glycerol this buffer was (25 mM

Tris•Cl, pH 7.5, 5%[w/v] glycerol). For Tris-chloride conditions without glycerol this buffer was (25 mM Tris•Cl, pH 7.5). For Tris-acetate conditions this buffer was (25 mM Tris•Acetate, pH 7.5). For Tris-glutamate conditions this buffer was (25 mM Tris•Glutamate, pH 7.5). For PIPES-KOH conditions this buffer was (20 mM PIPES•KOH, pH 6.8). Diluted nucleosomal arrays were incubated for 5 minutes at room temperature in these minimal chromatin dilution buffers before adding 1 volume of minimal phase separation buffer. For Tris-chloride conditions the predominant minimal phase separation buffer was (25 mM Tris•Cl, pH 7.5, 300 mM NaCl, 2 mM $MgCl_2$, 5%[w/v] glycerol). For Tris-acetate conditions the predominant minimal phase separation buffer was (25 mM Tris•Acetate, pH 7.5, 300 mM potassium acetate, 2 mM magnesium acetate). For Tris-glutamate conditions the predominant minimal phase separation buffer was (25 mM Tris•Glutamate, pH 7.5, 300 mM potassium glutamate, 2 mM magnesium glutamate). For PIPES-KOH conditions the predominant minimal phase separation buffer was (140 mM PIPES•KOH, pH 6.8, 2 mM $MgCl_2$, 2 mM EGTA). Supplements to buffer composition were added to these phase separation buffers, with BSA at 0.2 mg/mL; DTT at 10 mM; glucose at 40 mM; Glucose Oxidase and Catalase at 20 and 3.5 μg/mL (photo-crosslinking assays) or 2 and 0.35 μg/mL (all else), respectively. For varying concentrations of salt, phase separation buffers were adjusted to achieve the intended final concentration of salt indicated in figures, figure legends, or text. After adding phase separation buffer reactions were gently and thoroughly mixed and added to a PEGylated and BSA passivated (unless otherwise indicated) microscopy well using a cut pipet tip.

Unless noted differently (e.g., 10 nM nucleosome concentration), condensates throughout were formed at a final nucleosome concentration of 1 μM in a final buffer composition of 25 mM Tris•Acetate, pH 7.5, 150 mM potassium acetate, 1 mM magnesium acetate.

Partial Droplet FRAP. Nucleosomal arrays with 1 in 100 fluorophore-labeled histone H2B proteins were diluted to 2 μM nucleosome concentration into a minimal chromatin dilution buffer (see compositions outlined above). Diluted nucleosomal arrays were incubated for 5 minutes at room temperature before adding 1 volume of minimal phase separation buffer (see compositions outlined above). Supplements to phase separation buffer compositions outlined above, as indicated in text or Figure Legends, were BSA at 0.2 mg/mL; DTT at 10 mM; glucose at 40 mM; Glucose Oxidase and Catalase at 2 and 0.35 μg/mL, respectively. After adding phase separation buffer, reactions were gently and thoroughly mixed and added to an mPEGylated but not BSA passivated microscopy well using a cut pipet tip. 1 to 2 hours after condensate formation, the central third or less of the condensate by area was photobleached to ~50% pre-bleach fluorescence intensity using 488 nm light on a laser scanning confocal microscope. Fluorescence recovery after photobleaching was measured at regular intervals using time-lapse fluorescence confocal fluorescence microscopy.

Unless noted differently (e.g., 10 nM nucleosome concentration), condensates in FRAP assays were formed at a final nucleosome concentration of 1 μM in a buffer composition of 25 mM Tris•Acetate, pH 7.5, 150 mM potassium acetate, 1 mM magnesium acetate.

Whole Droplet FRAP. Nucleosomal arrays with 1 in 100 fluorophore-labeled histone H2B proteins were diluted to 200 nM nucleosome concentration into a Minimal Acetate Dilution Buffer (25 mM Tris•Acetate, pH 7.5). Diluted nucleosomal arrays were incubated for 5 minutes at room temperature before adding 1 volume of Whole Droplet FRAP Buffer (25 mM Tris•Acetate, pH 7.5, 300 mM potassium acetate, 2 mM magnesium acetate, 0.2 mg/mL BSA, 10 mM DTT). After adding FRAP buffer, reactions were gently and thoroughly mixed and added to an mPEGylated but not BSA passivated microscopy well using a cut pipet tip. 1 to 2 hours after condensate formation, the entirety of condensates were photobleached using 488 nm light on a laser scanning confocal microscope. Fluorescence recovery after photobleaching was measured at regular intervals using time-lapse fluorescence confocal fluorescence microscopy.

Spinning disc confocal microscopy. Photo-crosslinking and confocal fluorescence microscopy imaging were captured on a Nikon Eclipse Ti microscope base equipped with a Yokogawa CSU-X1 spinning disk confocal scanner unit, 100 X 1.49 NA objective, and Andor EM-CCD camera.

Laser scanning confocal microscopy. Beside photo-crosslinking assays, all confocal fluorescence imaging was performed using a Leica SP8 confocal fluorescence microscope equipped with a resonant scanning stage, 20x dry objective, EM-CCD camera, and FRAP module. FRAP was performed using a non-resonant line scanning stage.

Imaging Condensates in the Narlikar Lab.

Phase Separation of Nucleosomal Arrays. Nucleosomal arrays with 46 base pair internucleosome linker DNA length and no fluorophore label, assembled in the Narlikar lab, were diluted to 1 μM nucleosome concentration in a minimal chromatin dilution buffer. For Tris-chloride conditions with glycerol this buffer was (25 mM Tris•Cl, pH 7.5, 5%[w/v] glycerol). For Tris-acetate conditions this buffer was (25 mM Tris•Acetate, pH 7.5). For Tris-glutamate conditions this buffer was (25 mM Tris•Glutamate, pH 7.5). For PIPES-KOH conditions this buffer was (20 mM PIPES•KOH, pH 6.8). Nucleosomal arrays were incubated for 5 minutes at room temperature in these minimal chromatin dilution buffers before adding 1 volume of minimal phase separation buffer. For Tris-chloride conditions this buffer was (25 mM Tris•Cl, pH 7.5, 300 mM NaCl, 2 mM $MgCl_2$, 5%[w/v] glycerol). For Tris-acetate conditions this buffer was (25 mM Tris•Acetate, pH 7.5, 300 mM potassium acetate, 2 mM magnesium acetate). For Tris-glutamate conditions this buffer was (25 mM Tris•Glutamate, pH 7.5, 300 mM potassium glutamate, 2 mM magnesium glutamate). For PIPES-KOH conditions this buffer was (140 mM PIPES•KOH, pH 6.8, 2 mM $MgCl_2$, 2 mM EGTA). After adding phase separation buffer reactions were gently and thoroughly mixed, incubated for 30 minutes at room temperature, and added to a PEGylated and BSA passivated microscopy well using a cut pipet tip.

Bright-field Light Microscopy. Images were captured on a Widefield Nikon Ti inverted microscope base equipped with a Nikon DS-Qi2 monochrome camera and Plan Apo 40x/0.95 objective with 1.5x magnification booster. Time-

lapse images were recorded over 3 minutes at 1 second intervals across 1608x1608 pixels with a pixel resolution of 122 nm x 122 nm.

Imaging Condensates in the Gerlich Lab.

Phase Separation of Nucleosomal Arrays. Nucleosomal arrays with 25 bp internucleosome linker length and no fluorophore label, assembled in the Rosen lab, were diluted to 1 µM nucleosome concentration in Minimal Acetate Dilution Buffer (25 mM Tris•Acetate, pH 7.5) and incubated for 5 minutes at room temperature. Phase separation was induced by the addition of 1 volume of Minimal Acetate Phase Separation Buffer (25 mM Tris•Acetate, pH 7.5, 300 mM potassium acetate, 2 mM magnesium acetate) with and without supplement of 0.2 mg/mL BSA, 10 mM DTT, 40 mM glucose, 2 µg/mL Glucose Oxidase, and 0.35 µg/mL Catalase as indicated in the text. After adding phase separation buffer reactions were gently and thoroughly mixed and added to a PEGylated and BSA passivated microscopy well using a cut pipet tip.

Differential Interference Contrast Microscopy. After a 90-minute incubation, microscopy images were captured on an Axio Observer Z1/7 microscope equipped with a Plan-Apochromat 20x/0.8 objective and Hamamatsu Orca Flash 4 Camera at 2.2 Volts at 22-25˚C. Time-lapse images were recorded over 10 minutes at 0.4 second intervals with 10 millisecond exposures across 2048x2048 pixels with a pixel resolution of 325 nm x 325 nm.

Condensate Photocrosslinking Assay.

Magnesium-dependent Phase Separation of Nucleosomal Arrays. Nucleosomal arrays with a 25 bp internucleosome linker DNA length and 1 in 20 to 1 in 100 AlexaFluor 488-labeled histone H2B proteins were diluted to 1 µM nucleosome concentration in a Minimal Acetate Dilution Buffer (25 mM Tris•Acetate, pH 7.5). Diluted nucleosomal arrays were incubated for 5 minutes at room temperature in dilution buffer before adding 1 volume of Magnesium-dependent Phase Separation Buffer (25 mM Tris•Acetate, pH 7.5, 100 mM potassium acetate, 4 mM magnesium acetate) for a final buffer concentration of 25 mM Tris•Acetate, pH 7.5, 50 mM potassium acetate, and 2 mM magnesium acetate. Buffering supplements added as indicated to the Magnesium-dependent Phase Separation Buffer were BSA at 0.2 mg/mL; DTT at 10 mM; glucose at 40 mM; Glucose Oxidase at 20 µg/mL; Catalase at 3.5 µg/mL. After adding Magnesium-dependent Phase Separation Buffer, reactions were gently and thoroughly mixed and added to a PEGylated, but not BSA passivated, microscopy well using a cut pipet tip.

Photocrosslinking. One to two hours after addition to the well, condensates of varying were exposed to 20 to 110 W/cm^2 of 488 nm light for 500 or 50 milliseconds, as indicated, using a spinning disk confocal fluorescence microscope.

EDTA-dependent dissolution of non-crosslinked condensates. After fluorescent light exposure, 1 µL of 500 mM EDTA was added to 40 µL of solution in the microscopy well to dissipate condensates that were not crosslinked to one another.

Imaging photo-crosslinked condensates. One minute after the addition of EDTA, images were taken at the exact site of light exposure. If photocrosslinking occurred, as indicated by light-dependent persistence of EDTA-resistant condensates, images were taken adjacent to the crosslinked condensate scar (e.g., left, right, up, down).

Quantification and Statistical Analyses. Statistical tests performed on experimental data and their representations are noted in figure legends. Image analysis was performed using ImageJ (Version 1.53) (4). Unless otherwise described, equivalent brightness and contrast were used when depicting microscopy images in each panel. In general, microscopy data processed by ImageJ was graphed using the R Statistical Package(5).

Determining Inverse Capillary Velocity from Condensate Fusion.

Image Processing. In ImageJ, microscopy images were flatfield corrected using a gaussian blur and set to a threshold to find intrinsic chromatin condensates in each image. Particles were then analyzed, outputting a unique identifier, spatial information (e.g., XM, YM, Circularity, etc.), and Slice number for each condensate in each image.

Identifying condensate fusion events. Using a custom script within the R Statistical Package (available upon request), individual condensate tracks were generated across the time-lapse by finding the nearest condensate ($\leq 10\ \mu m^2$) between each frame. Bona fide fusion events were found among these condensate

tracks by identifying two condensate tracks that merge coinciding with a sudden increase condensate area and aspect ratio that decreases exponentially over time. The fidelity of many of the identified condensate fusion events were manually verified in microscopy images.

Calculating inverse capillary velocity. For each condensate fusion event, the characteristic relaxation time (τ) was extracted from fit of aspect ratio (AR) over time (t) to $AR = 1 + (AR_{init} - 1) \cdot e^{-t/\tau}$, where AR_{init} is the initial aspect ratio following the onset of fusion. The diameter of each condensate, before and after fusion, was calculated using the area output (in μm^2) from ImageJ. Inverse capillary velocity was then calculated from the linear fit of τ and the combined pre-fusion condensate diameters across two biological replicates. The mean and standard deviation of these replicates is represented graphically in this manuscript.

Determining Condensate Diameters Over Time.

Image Processing. In ImageJ, 5 or more microscopy images for each time point in each buffer were thresholded by fluorescence intensity to find chromatin condensates. Particles were then analyzed, outputting a unique identifier, spatial information (e.g., XM, YM, Circularity, etc.), and Slice number for each condensate in each image.

Calculating Condensate Diameters. Using the R Statistical package, the diameter for each identified condensate was calculated using the area output (in μm^2) from ImageJ. These diameters were then plotted as a notched boxplot and

the statistical differences between time points determined using the student's t-test.

FRAP Quantitation.

Image Processing. Image analysis was performed using ImageJ. Using unbleached condensates as a control, photobleaching was corrected across the time course using a ratio-metric technique.

Partial Droplet FRAP analysis. For each image in each condensate, a 20-pixel wide line plot was calculated across the condensate at the bleached locus. Partial droplet FRAP analysis was completed for each experiment using these line plots and a custom script in R (available upon request). For partial droplet FRAP of condensates composed of dodecameric nucleosomal arrays in different buffered salt solutions in Figure 2, fluorescence recovery within the bleached region was determined relative to post-bleach mean intensity. (Note: we had previously determined that these condensates mix internally over the course of fluorescence recovery (1)). For partial droplet FRAP of all other condensates, fluorescence recovery within the bleached region was determined relative to the normalized max intensity signal of each line plot. This computational strategy is used to measure for internal mixing after photobleach (i.e. return to homogeneity) independent of differences in fluorescent molecule influx from solution between constructs and solution conditions.

Whole Droplet FRAP analysis. Whole droplet FRAP was determined from the mean corrected fluorescence intensities of condensates before and after photobleach.

Quantitation of Condensate Movement.

Image Processing. In ImageJ, condensates were identified across 4 technical replicates per condition from 2-minute-long time-lapse microscopy acquisition of 500 milliseconds per frame. For each time point in each condition and each replicate images were set to a threshold by fluorescence intensity to find chromatin condensates. Particles were then analyzed, outputting a unique identifier, spatial information (e.g., XM, YM, Circularity, etc.), and Slice number for each condensates in each image.

Condensate Tracking. Using a custom script in R (available upon request), individual condensate tracks were generated across the time-lapse by finding the nearest condensate ($\leq 10\ \mu m^2$) between each frame. Custom-generated graphical depictions of condensate-centered and relative trajectories can be found in Figure 4.

Calculating mean squared displacement versus lag time. Condensate tracks were then segregated into 15 second segments, with 30 condensate positions per track per segment, for each 500-millisecond window. The mean squared displacement between the initial position of each segment and the lagged time positions thereafter was determined. Mean squared displacement of from all segments from all condensates between 4 and 8 microns in diameter were used

to calculate the mean squared displacement versus lag time and diffusion coefficient for each condensate.

Data Availability. Datasets and software are available by requests to the corresponding author.

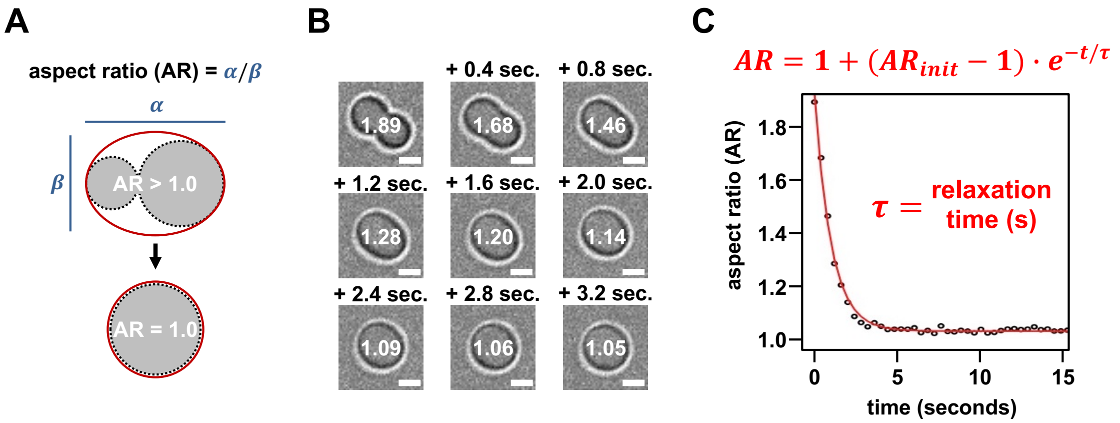

Figure S1. Quantitating condensate fusion rate. (A) Schematic depicting the calculation of aspect ratio (AR) during condensate fusion. (B) Time-lapse differential interference contrast microscopy of a rounding condensate following fusion along with the calculated aspect ratio determined for each acquisition. (C) Dot plot and exponential fit (red line) of a condensate fusion event from Figure S1B. Scale bars, in white, are 4 µm.

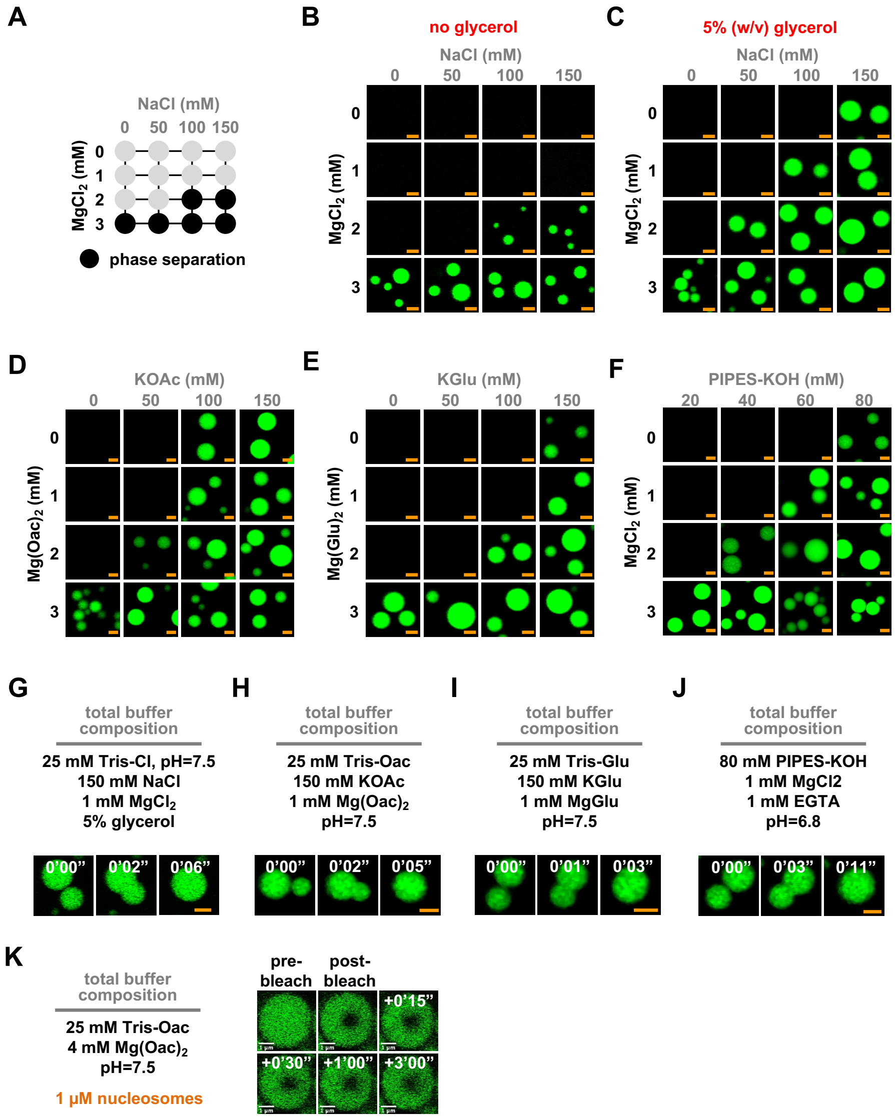

Figure S2. Phase separation of chromatin and fluor-labeled condensate fusion. (A) Phase diagram of chromatin in varying concentrations of NaCl and $MgCl_2$ in Tris-chloride buffer without glycerol. Dark circles indicate the presence of condensates. Confocal fluorescence microscopy images of intrinsic chromatin condensates composed of AlexaFluor 488-labeled arrays, in green, in varying concentrations of salt in (B) Tris-chloride buffer without glycerol, (C) Tris-chloride buffer with glycerol, (D) Tris-acetate, (E) Tris-glutamate, and (F) PIPES-KOH. With materials produced and experiments performed in the Rosen lab, time-lapse confocal fluorescence microscopy images of intrinsic chromatin condensates composed of AlexaFluor 488-labeled arrays, in green, undergoing condensate fusion in the indicated (G) Tris-chloride, (H) Tris-acetate, (I) Tris-glutamate, and (J) PIPES-KOH based buffered salt solutions. (K) Partial droplet FRAP recovery of intrinsic chromatin condensates formed in a buffer with 25 mM Tris-acetate, and 4 mM magnesium acetate at 1 μM nucleosome concentration of AlexaFluor 488-labeled arrays, in green. Scale bars, in orange and white, are 4 and 1 μm, respectively.

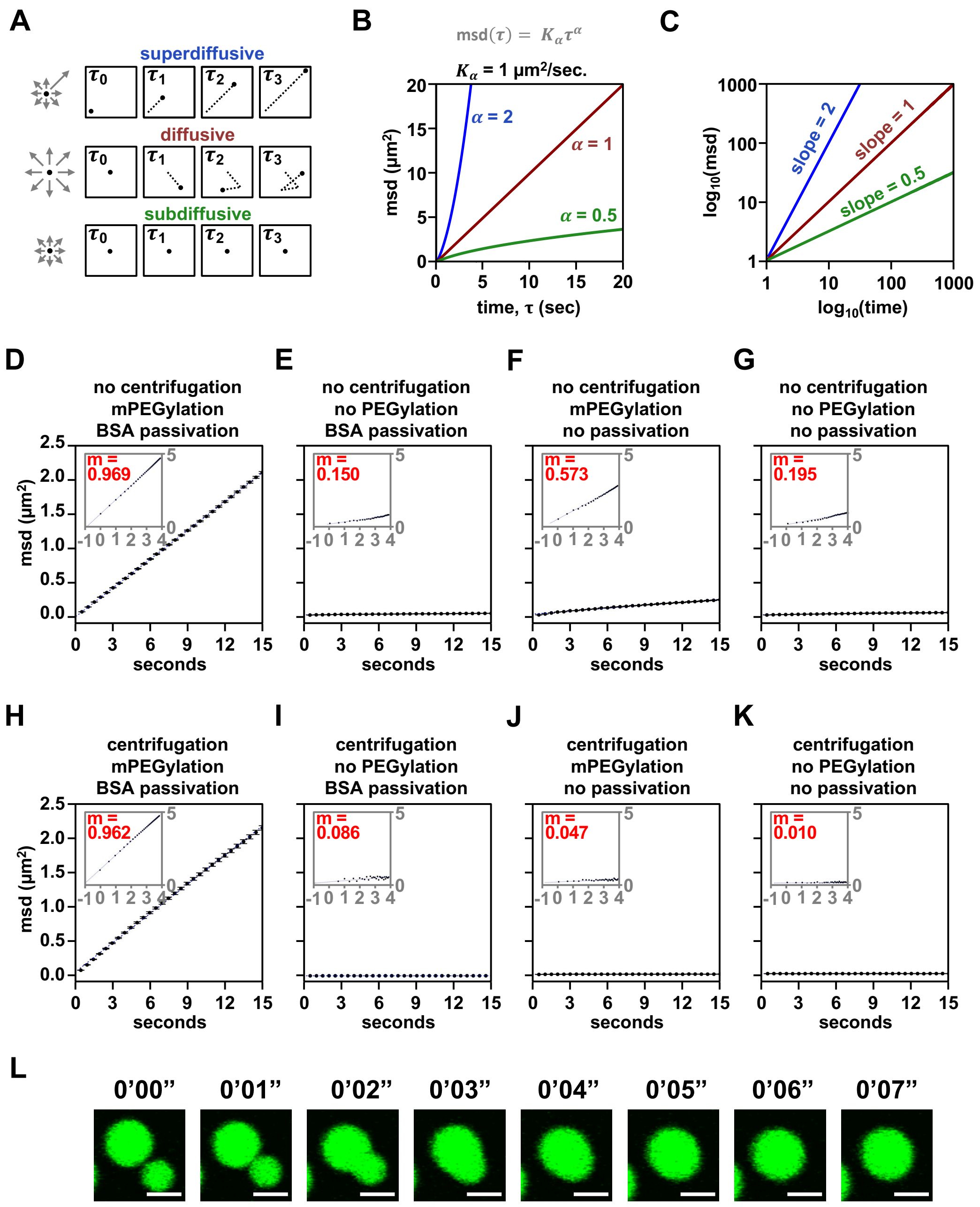

Figure S3. Motion of condensates with differing sample preparation and condensate fusion without BSA. (A) Graphical depiction of different kinds of particle motion. (B) Plot and (C) log-transformed plot of particle motion over time given different parameters for K_α and α. (D-K) Plot of chromatin condensate motion over lag time with log transformation inset and slope of log-log plot in red. (L) Time-lapse confocal fluorescence microscopy images of intrinsic chromatin condensates composed of AlexaFluor 488-labeled arrays, in green, undergoing fusion without BSA passivation. Scale bars, in white, are 4 μm.

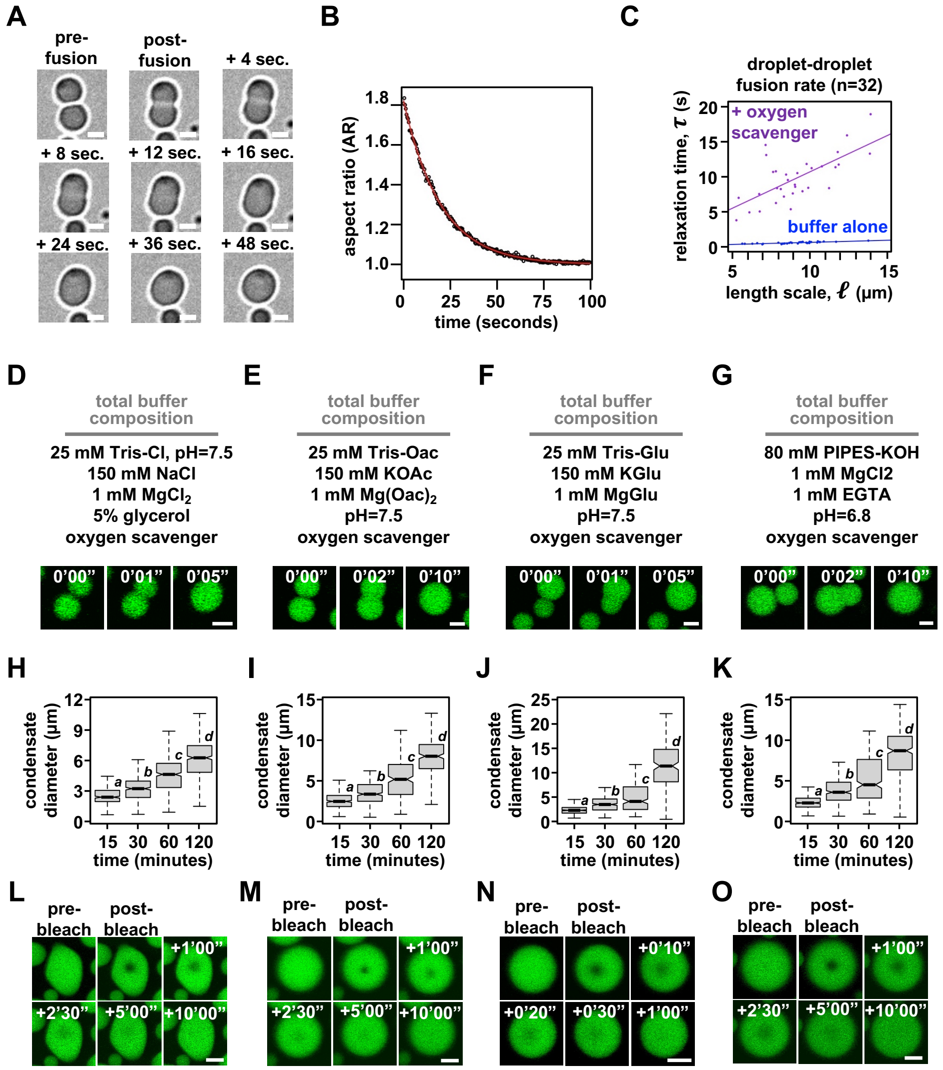

Figure S4. Condensate dynamics and properties in the presence of an oxygen scavenging system. (A) Time-lapse differential interference contrast microscopy of intrinsic chromatin condensates formed in the presence of oxygen scavenging components rounding after fusion. (B) Dot plot and exponential fit (red line) of a condensate fusion event from Figure S4A. (C) Relaxation time versus length scale (sum of pre-fusion diameters) for 32 individual instances of condensate fusion in the buffer composition indicated in Figure 1B, in blue, and that buffer containing oxygen scavenging components, in purple. Confocal fluorescence microscopy images of condensate fusion in (D) Tris-chloride, (E) Tris-acetate, (F) Tris-glutamate, and (G) PIPES-KOH buffers containing oxygen scavenging components. Boxplots of intrinsic chromatin condensate diameters following induction of phase separation in (H) Tris-chloride, (I) Tris-acetate, (J), Tris-glutamate, or (K) PIPES-KOH based buffers containing oxygen scavenging components. Bars marked with different letters are significantly different from one another (student's t-test, $p < 1x10^{-7}$). Fluorescence microscopy images of partial droplet FRAP of intrinsic chromatin condensates, in green, composed of nucleosomal arrays labeled with AlexaFluor 488 in (L) Tris-chloride, (M) Tris-acetate, (N), Tris-glutamate, or (O) PIPES-KOH based buffers containing oxygen scavenging components. Scale bars, in white, are 4 μm.

SI References

1. B. A. Gibson *et al.*, Organization of Chromatin by Intrinsic and Regulated Phase Separation. *Cell* **179**, 470-484.e421 (2019).
2. K. Luger, T. J. Rechsteiner, T. J. Richmond, Expression and purification of recombinant histones and nucleosome reconstitution. *Methods Mol Biol* **119**, 1-16 (1999).
3. S. Sanulli *et al.*, HP1 reshapes nucleosome core to promote phase separation of heterochromatin. *Nature* **575**, 390-394 (2019).
4. C. A. Schneider, W. S. Rasband, K. W. Eliceiri, NIH Image to ImageJ: 25 years of image analysis. *Nat Methods* **9**, 671-675 (2012).
5. R. C. Team, R: A language and environment for statistical computing. (2013).
